# Forest Management and the Colonization of Artificial Tree Holes by Aquatic Insect Larvae

**DOI:** 10.1002/ece3.71962

**Published:** 2025-08-15

**Authors:** Heidi Bartel, Martin M. Gossner, Jana S. Petermann

**Affiliations:** ^1^ Department of Environment and Biodiversity, Faculty of Natural and Life Sciences Paris Lodron University of Salzburg Salzburg Austria; ^2^ Forest Entomology Swiss Federal Research Institute WSL Birmensdorf Switzerland; ^3^ Department of Environmental Systems Science, Institute of Terrestrial Ecosystems ETH Zurich Zurich Switzerland

**Keywords:** aquatic insect larvae, biodiversity exploratories, colonization, community composition, land‐use intensity, tree hole analogues

## Abstract

Human activities in forests lead to alteration or even destruction of habitats for numerous organisms, often resulting in a decline of biodiversity. Insects inhabiting water‐filled tree holes may be especially sensitive to human impact as they require these microhabitats for at least part of their life cycle, with larvae mainly feeding on plant and animal debris accumulating in the water until they actively disperse in their adult stage. The processes leading to successful colonization of these microhabitats are not well understood, and it is unclear how forest management could influence them. We used sequential collection and recording of larval communities in artificial tree‐hole analogues to study the process of colonization by aquatic tree‐hole insects. We focused on the effects of parameters related to forest management as well as microhabitat properties on abundance, species richness, and community composition during colonization of artificial tree holes by aquatic tree‐hole insects. We observed complex, and partly species‐specific, temporal patterns of colonization of these new microhabitats. We found that the forest management intensity index ForMI, tree composition of forests as well as distance to natural water‐filled tree holes and debris type were important in shaping community composition of insect larvae inhabiting tree holes across the entire colonization process. Larval abundance was negatively affected by increased distance to natural microhabitats and by changes in microclimate. Our results suggest that forest management significantly impacts microhabitat colonization dynamics of tree‐hole insects, emphasizing the need for less‐intensively managed forests to support natural tree‐hole communities. We recommend the protection, creation, and maintenance of tree‐related microhabitats, for example, through the promotion of habitat trees in managed forests, to sustain higher abundances of tree‐hole inhabitants. Our findings underline the ecological value of water‐filled tree holes and support their integration into forest conservation strategies as both essential habitats and valuable indicators of environmental change.

## Graphical abstract

The present study demonstrates the effects of forest management on insect communities during the colonization of artificial tree holes and reveals a dynamic, species‐specific pattern of colonization. It highlights the negative effects of forest management intensity and the dominant tree species of the forest stand on insect abundance and community composition, in addition to the influence of dispersal distances and physico‐chemical water parameters. The study underscores the role of suitable forest management practices in preserving water‐filled tree holes and supporting biodiversity in forests.

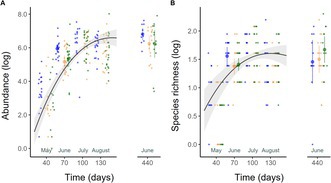

## 1 Introduction

Human impact, such as forest management, is considered to be one of the main global drivers of biodiversity loss (Newbold et al. 2015; Partel et al. 2025; Sala et al. 2000). Forest management practices can, for example, alter the age structure of forests and modify tree species composition, often resulting in a reduction of structural complexity. Together with forest fragmentation, this can have severe consequences for habitat and resource availability for a diverse range of organisms, such as bryophytes, lichens, fungi, and forest‐dwelling beetles (Paillet et al. 2010).

Tree‐related microhabitats, such as water‐filled tree holes, have been identified as key structures for forest biodiversity (Asbeck et al. 2021; Basile et al. 2020; Larrieu et al. 2018). Water‐filled tree holes are colonized by animals, protists, fungi, and other microorganisms and are particularly important for a wide variety of insect species that require aquatic habitats for their larval stages. Some of them are even specifically associated with tree holes and occur rarely or never in other aquatic habitats (Rohnert 1951). The influence of forest management practices on these small, isolated aquatic ecosystems in temperate forests has not often been addressed despite a long history of research on tree holes (Petermann and Gossner 2022). Forest management can reduce the availability of water‐filled tree holes, directly through tree harvesting and indirectly through the alteration of tree species composition. In Europe, fast‐growing species like spruce (*Picea albies*) are planted far outside their natural distribution ranges to increase timber production. This has caused substantial changes in forest structure, stand age, resource availability, and microclimatic conditions (Carnus et al. 2006). The few existing studies on the influence of forest management on insect communities associated with water‐filled tree holes revealed multiple indirect negative effects (Gossner et al. 2016; Petermann et al. 2020, 2016). For example, with increasing forest management, insect abundance and richness in water‐filled tree holes decreased significantly (Gossner et al. 2016; Petermann et al. 2016) which could partly be explained by reduced tree‐hole density, changes in debris accumulation, and changes in water chemistry (Gossner et al. 2016; Petermann et al. 2020, 2016). Furthermore, fragmentation and deforestation of forests lead to the isolation of patches, which restricts the movement of insect species between the remaining forest fragments (Khazan et al. 2015). This may reduce genetic exchange and genetic diversity, as shown in a recent meta‐analysis for many non‐tree‐hole species globally (Shaw et al. 2025).

Microhabitat‐related parameters, for example, tree‐hole size, height of tree holes on the tree, physico‐chemical water parameters, and the amount of debris often have been shown to have effects on abundance, species richness, and/or community composition (see Petermann and Gossner 2022). Water volume in tree holes as a measure of tree‐hole size was generally found to be positively related to abundance and species richness and to cause a shift in community composition (Petermann and Gossner 2022). The height of water‐filled tree holes on the tree is also important for structuring insect communities, with different responses shown for individual species (Gossner and Petermann 2022; Petermann et al. 2020). The effects of physico‐chemical water parameters, among them water temperature, dissolved oxygen content, pH, and conductivity, on community attributes have been analyzed in numerous studies (Devetter 2004; Gossner et al. 2016; Paradise 1998; Ptatscheck and Traunspurger 2015; Schmidl et al. 2008; Yanoviak et al. 2006). Modulating effects of pH and dissolved oxygen were found, but these were strongly taxon‐ or species‐specific (Bradshaw and Holzapfel 1988; Gossner and Petermann 2022; Paradise 1998; Paradise and Dunson 1997; Yoshida et al. 2018). For example, the mosquito larvae of *Aedes geniculatus* are commonly found in tree holes exhibiting high oxygen saturation and high pH, whereas the larvae of the scirtid beetle *Prionocyphon serricornis* occur more frequently in low‐oxygen conditions and tolerate lower pH values (Schmidl et al. 2008).

The aquatic larvae in tree holes feed largely on plant debris and animal remains that accumulate therein until they disperse in the adult stage to actively search for locations to lay their eggs (Petermann and Gossner 2022). Quite a few studies investigated the effects of nutrient availability on insect communities in tree holes (Carpenter 1983; Gossner et al. 2016; Paradise et al. 2008; Petermann et al. 2020). In general, insect community composition and food web structure were found to change in response to altered nutrient cycles in tree holes and other lentic systems (Gossner et al. 2016; Petermann et al. 2016; Pintar and Resetarits 2017). The meta‐analysis of Petermann and Gossner (2022) showed an overall positive relationship between abundances and species richness and debris amount. Gossner et al. (2016) showed that forest management, possibly through reduced debris input, shifts community composition towards filter feeders, while negatively impacting specialists and predators, leading to a reduction in the functional size of food webs. Studies concerning nutrient quality and variability in tree holes concentrated mainly on mosquito larvae and different hardwood leaves (Muturi et al. 2012; Walker and Merritt 1988; Yanoviak 1999). No data is available for conifer needles as a nutrient source for tree hole inhabiting insect larvae, but research from other lentic and lotic aquatic systems showed that the breakdown rate of conifer needles compared to leaves of deciduous trees was lower, resulting in lower densities of macroinvertebrates (Hisabae et al. 2011; Sedell et al. 1975). This distinction between debris types further influenced the dynamics of invertebrate colonization: habitats containing hardwood leaves were shown to be initially colonized at higher rates than those containing slower decomposing conifer needles, but this pattern reversed later on (Pintar and Resetarits 2017; Rosset and Bärlocher 1982).

Dispersal, arrival, and establishment are interdependent processes in the colonization of new habitats (Maguire 1971). To our knowledge, little is known about the dispersal and habitat selection behavior of the adult insects colonizing new water‐filled tree holes. Oviposition preferences of some mosquito, midge, and scirtid species are known to be influenced by tree‐hole size and the associated availability of food, as well as by chemical water characteristics (Bradshaw and Holzapfel 1988; Paradise 1998; Paradise and Dunson 1997; Schmidl et al. 2008). Different larval development times, sensitivity to predation, and competition are important parameters influencing the subsequent establishment of species in new habitats (Maguire 1971).

While a number of studies investigated the temporal dynamics of individual tree hole‐inhabiting taxa or species (see Gossner 2018), there is only a limited number of quantitative studies describing temporal dynamics of whole insect communities in temperate forests over more than one season. The latter mainly concentrated on the role of seasonal events of the environment such as drought or rainfall, abiotic factors, or food resources on temporal dynamics (Harlan and Paradise 2006; Paradise et al. 2008; Gossner 2018), but little attention has been given to colonization processes over time (Paradise et al. 2008; Ptatscheck and Traunspurger 2015).

In the present study, we use artificial tree holes as a model system in three temperate forests in Germany, as they are replicable and provide the possibility to conduct sequential harvests, to achieve the following three objectives:

1. To investigate the temporal pattern of colonization of these microhabitats by larval aquatic insect communities. We sampled over one complete season to cover a full seasonal cycle and did an additional sampling 15 months after setup of the experiment to study the longer‐term colonization. We hypothesize that tree hole analogues will be rapidly colonized by larval aquatic insects following seasonal fluctuation of both abundance and species richness. We expect insect abundances to increase in the following season due to a longer period available for colonization, which may allow even less dispersive species to establish. Additionally, enhanced nutrient availability from decomposing debris could support increasing abundances with time.
2. To test the effects of parameters related to forest management, that is, forest management intensity, dominant tree species, and distance to the nearest natural water‐filled tree hole, on abundance, species richness, and community composition of aquatic insect larvae in general, and the colonization patterns of water‐filled tree holes in particular. We hypothesize that colonization decreases with increasing forest management intensity. This effect may partly be driven by alterations in tree composition towards more conifer‐dominated stands that are less likely to form water‐filled tree holes, reducing the availability of suitable microhabitats and increasing the distances between remaining tree holes.
3. To test the effects of microhabitat properties, that is, debris type, aspect (northness) as a proxy for sun exposure and thus temperature, water volume, and chemical water parameters on abundance, species richness, and community composition of aquatic insect larvae, and the colonization patterns of water‐filled tree holes. We hypothesize that microhabitat properties, particularly nutrient quality, play a significant role in shaping colonization patterns.

## 2 Material and Methods

### 2.1 Study Regions and Sites

Our study was conducted in three regions of the Biodiversity Exploratories (Fischer et al. 2010) distributed across Germany: the UNESCO Biosphere Reserve Schwäbische Alb (henceforth “Alb”), the National Park Hainich and the surrounding Hainich‐Dün forest (henceforth “Hainich”), and the UNESCO Biosphere Reserve Schorfheide‐Chorin (henceforth “Schorfheide”). Alb is located in southwestern Germany (48°34′–48°53′N; 9°18′–9°60′E) at an altitude of 460–860 m above sea level. The annual average temperature is 6°C–7°C and precipitation is 700–1000 mm per year. The landscape is characterized by a mosaic of grassland and forests, ranging from unmanaged beech forests that were used as woodland pastures in the past and are now protected as nature reserves to mixed forests to even‐aged beech forests and intensively managed spruce monocultures. Hainich is a forested hill chain in central Germany (50°94′–51°38′N; 10°17′–10°78′E) at an altitude of 285–550 m. It receives 500–800 mm of rain per year and has an annual mean temperature of 6.5°C–8°C. Hainich includes the largest unfragmented area of hardwood forests in Germany. Only 12% of total forest area is covered by conifers (spruce). Hainich shows a wide spectrum of forest management ranging from unmanaged forests in the National Park Hainich (20–70 years since last management) to uneven‐aged and even‐aged beech forests to spruce monocultures. Schorfheide is located in northeastern Germany (52°51′–53°11′N; 13°36′–14°01′E) at an altitude of 3–140 m above sea level. It is the driest of the three regions with an annual precipitation of 500–600 mm and a mean annual temperature of 8°C–8.5°C. The forest area comprises intensively managed pine monocultures, managed mixed pine‐beech forests, managed oak forests, and even‐aged managed and unmanaged beech forests. The distances between Alb and Hainich and Hainich and Schorfheide are approximately 300 km. A more precise description of forests and study plots is provided by Fischer et al. (2010), Boch et al. (2013), and Schall et al. (2018).

From each of the three exploratories, 12 plots were selected from a pool of 50 forest experimental plots, each 1 ha in size, resulting in a total of 36 plots included in the study. These represent the main three forest management types: four plots were unmanaged beech forest, four plots were uneven‐ and even‐aged managed beech forests, and four plots were conifer monocultures, that is, spruce in Alb and Hainich. For quantifying the effects of forest management on artificial tree holes and their invertebrate communities, we used the forest management intensity index (ForMI) proposed by Kahl and Bauhus (2014). ForMI reflects management activities over the past 30–40 years and is calculated as the sum of three management‐related factors, each ranging from 0 (no management) to 1 (intensive management): *Iharv* represents the proportion of harvested volume relative to the total volume of standing, harvested, and dead wood volume. *Inonat* measures the proportion of trees that are not part of the natural vegetation at the sites based on the volume of harvested, living, and dead wood (without saw cuts) of non‐natural tree species in relation to the volume of all tree species. *Idwcut* measures the proportion of dead wood volume with saw cuts relative to the total amount of dead wood volume. In our study plots, ForMI values ranged from 0 to 2.4 (mean 1.1 ± SD 0.7). Forests dominated by beech trees showed ForMI values ranging from 0 to 1.2 (mean 0.7 ± SD 0.4) whereas conifer‐dominated forests showed higher values: forests dominated by pine ranged from 1.4 to 1.9 (mean 1.6 ± SD 0.2) and spruce‐dominated forests from 1.7 to 2.4 (mean 2.1 ± SD 0.2).

### 2.2 Experimental Design

We set up a total of 360 artificial tree holes on the 36 plots (10 per plot) between 23 March and 20 April 2015. On each plot, 10 containers were placed on two individual trees of approximately the same DBH (diameter at breast height; 0.8–2.4 m (mean 1.56 ± 0.014 m SD)), and with a distance of 9–72 m (mean 31 ± 12 m SD) between them. A constant height of about 3 m was chosen to minimize the effect of vertical stratification on insect colonization and to protect the traps from accidental disturbance by animals and people. On each tree, five uniquely numbered artificial tree holes were distributed evenly in a circle around the tree trunk without aligning them to a specific cardinal direction and secured using coated wiring (FE/PP STABILIT, 1.0 mm, BAHAG AG, Mannheim, Germany) (Figure S1). The trees were selected to allow all containers to be shaded by the canopy. The distance to the nearest natural water‐filled tree hole was determined based on mapping conducted in a previous study (Petermann et al. 2020) to assess the effect of sources of potential colonization.

As artificial tree holes, we used brown plant pots with a maximum volume of 2.5 L. The inside of these containers was partially lined with window screen (Mako Easy Life GmbH, mesh size 1.2 mm) to allow scirtid beetles movement along the walls of the container (Paradise and Kuhn 1999). To prevent additional leaf litter from entering the artificial tree holes a standard window insect screen was placed as an inverted funnel about 5–10 cm above the artificial tree hole. A hole in the center of the screen allowed the adult insects to escape (Figure S1). Rainwater, natural tree twigs as well as leaves from either beech or conifer trees had been collected earlier in each region, that is, beech leaves in each exploratory region, spruce needles in Alb and Hainich and pine needles in Schorfheide. Rainwater was boiled and filtered, and the twigs were heat sterilized to prevent microbial contamination and ensure consistency across the experiment. Leaves were partly cut to simulate coarse and fine detritus fractions typically present in natural tree holes, litter was washed using tap water, heat sterilized for 2 days at 70°C, and then placed in distilled water for 2 days prior to experiments. The containers were filled with 1.7 L of sterilized rainwater from the corresponding region, a twig was inserted to allow insect movement and to serve as a perch for ovipositing insects, and debris was added. All containers on one tree contained 6.5 g dry weight of beech leaf debris, and on the other tree they contained 6.5 g conifer debris. Before the artificial tree holes were placed on the tree, initial water temperature, dissolved oxygen, and pH were measured (Hach HQ40D.99.101301 digital multi meter kit) in one container from each debris' type. Initial pH ranged from 6.46 to 8.04 (mean 7.33 ± SD 0.33). Every 4 weeks from the start of the experiment containers were refilled with rainwater to the original level to ensure that all containers continuously contained water.

### 2.3 Sampling of Organisms

We conducted a full‐season sampling by collecting one artificial tree hole per tree at about 4‐week intervals between 11 May and 25 August 2015. This was followed by a single sampling in June 2016, approximately 15 months after installation. This approach ensured sufficient temporal resolution while remaining practically feasible. Each container was sampled only once throughout the study to ensure independent data points. Each container was reinstalled after collecting its contents in order to keep the design constant. Oxygen content, temperature, and pH were measured with a multiprobe (Hach HQ40D.99.101301 digital multi meter kit) prior to the collection of the samples. The entire contents of artificial tree holes were transferred to closed labeled containers during sampling, taken to the laboratory, and stored at 4°C–8°C until sorting. After separation of the coarse debris (leaves, conifer needles etc.) by means of a 0.5 mm sieve and measurement of the actual amount of water in the lab, all aquatic invertebrates were picked from debris and water and transferred to 99.6% ethanol. Fine debris was filtered from the water using a filter with a pore size of 12.5 μm. Debris was dried at 70° for 72 h, and dry weight was determined. Where possible, the insect larvae were identified to species level under a stereomicroscope using insect larval identification keys (Cranston et al. 1987; Klausnitzer 1996; Nilsson 1997; Rotheray 1993; Smith 1989; Sundermann et al. 2007; Thyssen 2010). All other insect larvae were assigned to morphospecies (based on Gossner et al. 2016; Petermann et al. 2016). To verify morphological identification, we conducted DNA barcoding on the most common larval species. Eggs, dipteran pupae, non‐aquatic arthropods, and non‐arthropods (e. g. gastropods, annelids) were also collected, but not included in statistical analysis.

## 3 Statistical Analysis

All statistical analysis was performed in R version 4.3.2 (R Core Team 2023). We analyzed the effect of region and explanatory variables related to management decisions at plot scale, that is, forest management intensity index (ForMI), dominant tree species, and distance to the nearest natural water‐filled tree hole on several response variables: total abundance of insect larvae, (morpho)species richness, community composition, and the abundance of the most abundant species, which were also the most frequently found species in artificial tree hole communities. Additionally, explanatory variables at artificial tree hole scale were used in the same models, that is, type of debris inserted in containers (beech vs. spruce/pine), time, aspect (northness), as well as water volume and chemical water parameters (dissolved oxygen and pH) at the time of sampling of the individual artificial tree hole. Time was calculated as days since the container was deployed, and northness was derived by converting directional data into radians and applying the cosine function, resulting in values from −1 (south‐facing) to 1 (north‐facing). In a first step, Spearman correlations were calculated between all explanatory variables with the aim to exclude highly correlated variables from further analysis. The only strong correlation that emerged was between ForMI and the dominant tree species (Spearman correlation coefficient = 0.74, see Figure S2). Because of this correlation, we ran two separate linear mixed effects models including either forest management or dominant tree species besides the above‐mentioned explanatory variables using the function *lme* in R package “nlme” (Pinheiro, Bates, and R Core Team 2023). Model validation showed better AICc values for the model including ForMI; thus, the results from this model are reported in the main text, while the results of the model including dominant tree species are provided in the Data S1. We incorporated also second and third order polynomials of time expressed as days since setup of the containers, yielding a better fit of the models due to non‐linear effects of time. This approach captures the expected pattern, including initial colonization, a peak in abundance and/or species richness in summer, followed by a decline due to insect emergence and a subsequent increase in the following season due to time‐lagged colonization by less dispersive species and increased nutrient availability through decomposition, which promotes further colonization and community development. Selected two‐way interactions between explanatory variables were also included in the models to test our hypotheses. These were the interaction between forest management (ForMI) or dominant tree species and debris type, as well as the linear effect of time, and interactions between distance to the nearest natural tree hole and all tree hole scale variables (i. e. debris type and the water parameters water volume, dissolved oxygen, and pH at the time of sampling) and time. Abundance and species richness data were log‐transformed to meet the assumptions of the models. Additionally, abundance (log transformed) was incorporated as a covariate in the analysis of species richness. In all models, individual tree ID nested in plot was specified as a random effect. We used the *effects* function available in R package “effects” (Fox and Weisberg 2018a, 2018b) to compute the effects of the predictors and created the graphs with *ggplot* from the R package “ggplot2” (Wickham 2016).

To visualize the differences in community composition, non‐metric multidimensional scaling (NMDS) plots were produced with the *metaMDS* function available in the R package “vegan” (Oksanen et al. 2022) with a maximum of 1000 random starts and three dimensions. Artificial tree holes without any insect larvae and those containing only one species were excluded from the NMDS analysis. Based on the same subsample (excluding all zero‐ and 1‐species communities) PERMANOVAs (function *adonis2* in “vegan”; Oksanen et al. 2022) on Bray‐Curtis matrices using a permutation scheme with 9999 permutations, allowing permutation within tree and blocking permutation across plots, were run to test the effect of explanatory variables on community composition. Using the same approach as for the mixed models, we included the following explanatory variables: region, ForMI or dominant tree species, distance to the nearest natural water‐filled tree hole, the type of debris inserted in containers, time (including second and third order polynomials), northness, water volume, dissolved oxygen, pH, and the two‐way interactions described above.

## 4 Results

We examined 321 of the 360 containers originally installed in the regions Alb, Hainich, and Schorfheide throughout the five sampling times (monthly May to August 2015 and additionally in June 2016). Thirty‐nine containers were lost due to felling of trees (*n* = 15), breakage of containers (*n* = 12) or other reasons (*n* = 12). Thirteen artificial tree holes did not contain any insect larvae, all of them during the first sampling after one month. In the remaining 308 artificial holes, a total of 143,839 insect larvae that represent 41 species and morphospecies of Diptera, and one species of Coleoptera (Scirtidae: *Prionocyphon serricornis*; hereafter referred to as species) were collected. Twenty‐three individuals that could not be identified due to the very bad condition of the body were excluded from the statistical analysis.

The total abundance of insect larvae varied between the three regions, being marginally significantly higher in Hainich and Schorfheide than in Alb in all sampling periods (Table 1 and Tables S1 and S3). Species richness was significantly related to abundance but did not show differences between the regions with or without abundance as a covariate (Table 1 and Tables S1–S3).

**TABLE 1:** Results of the lme model (abundance, species richness) and PERMANOVA (community composition) testing the effect of explanatory variables and two‐way interactions on abundance, species richness and composition of the communities. Abundance and species richness were log‐transformed prior to the analysis. Abundance (log‐transformed) is used as a covariate in the analysis of species richness. Results of the model without abundance as a covariate are shown in the Table S2. In the lme models individual tree number nested in plot was specified as a random effect. In the PERMANOVA, plot was specified as blocking factor for permutation tests using the blocks argument. Significant *p*‐vales (< 0.05) are shown in bold, while 0.05 < *p* < 0.1 are shown in italic. Arrows indicate the positive (↑) or negative (↓) effects of numeric variables on response variables. ForMI = Forest management intensity index. Estimated variances for the random effects: Plot 9.3e‐09, 6.09e‐14, tree within plot 0.018, 3.09e‐05.

		*Abundance*				*Species richness*				*Community composition*		
	ndf	ddf	*F*‐value	*R* ^2^	*p*	ddf	*F*‐value	*R* ^2^	*p*	*F*‐value	*R* ^2^	*p*
Abundance	1					174	146.97	0.070	**< 0.0001**			
Region	2	28	2.83	0.170	*0.0762*	28	1.00	0.027	0.3821	3.73	0.025	**0.0001**
ForMI	1	28	3.64	0.004	*0.0667* ↓	28	1.44	0.014	0.2396	4.85	0.017	**0.0001**
Distance to nearest natural tree hole (m)	1	27	13.76	0.025	**0.0009** ↓	27	0.14	0.006	0.7123	2.15	0.007	**0.0001**
Debris type	1	27	1.58	0.006	0.2202	27	0.03	0.000	0.8700	1.27	0.004	**0.0001**
Time (days since set‐up)	1	175	91.90	0.277	**< 0.0001**	174	0.34	0.059	0.5582	16.96	0.058	**0.0001**
Time (second order polynomial)	1	175	159.49	0.175	**< 0.0001**	174	4.41	0.066	**0.0371**	20.96	0.071	**0.0001**
Time (third order polynomial)	1	175	47.46	0.126	**< 0.0001**	174	14.02	0.054	**0.0002**	10.73	0.037	**0.0001**
Northness		175	4.47	0.004	**0.0359** ↓	174	3.38	0.010	*0.0678*	1.28	0.004	0.2604
Water volume (ml)	1	175	11.86	0.057	**0.0007** ↓	174	1.58	0.001	0.2112	2.50	0.009	*0.0576*
Dissolved oxygen (mg/l)	1	175	1.10	0.034	0.2948	174	1.09	0.006	0.2988	1.18	0.004	0.2557
pH	1	175	1.03	0.023	0.3106	174	0.17	0.023	0.6823	0.29	0.001	0.9358
ForMI:Debris type	1	27	0.03	0.001	0.8549	27	0.43	0.002	0.5160	0.88	0.003	**0.0001**
ForMI:Time	1	175	0.43	0.001	0.5138	168	1.14	0.003	0.2277	0.28	0.001	0.9721
Distance to nearest natural tree hole:Time	1	175	0.28	0.002	0.5988	168	1.47	0.007	0.2270	0.93	0.003	0.3989
Debris type:Time	1	169	0.06	0.000	0.8022	168	2.47	0.006	0.1180	0.59	0.002	0.7009
Water volume:Time	1	169	2.88	0.015	*0.0918*	168	0.88	0.002	0.3493	1.20	0.004	0.2471
Dissolved oxygen:Time	1	169	13.29	0.03	**0.0004**	168	0.06	0.005	0.8008	2.41	0.008	*0.0561*
pH:Time	1	169	2.81	0.011	*0.0957*	168	8.17	0.031	**0.0048**	0.42	0.001	0.8957

### 4.1 Temporal Patterns of Abundance and Species Richness

**FIGURE 1:**
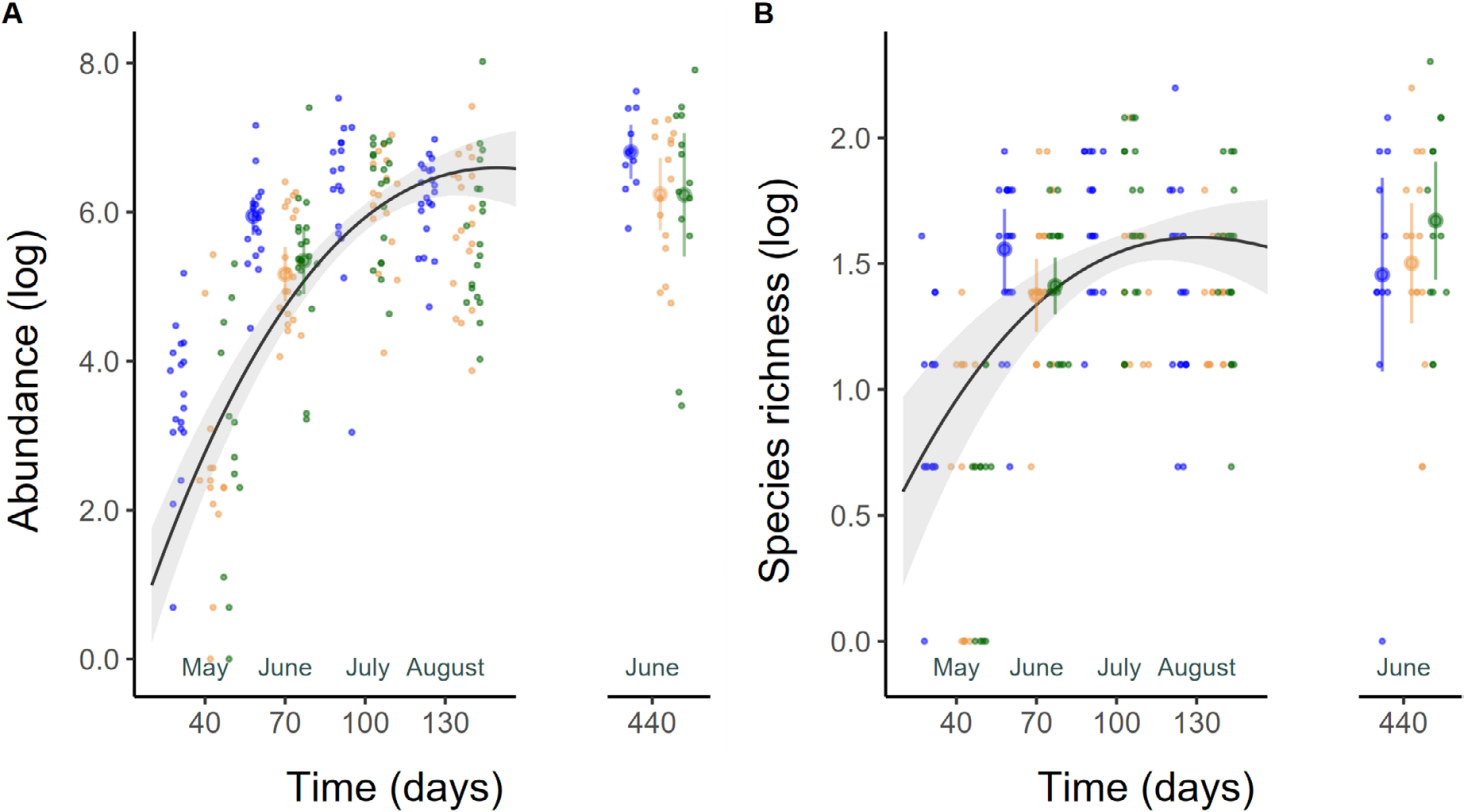
Temporal patterns of abundance (A) and species richness (B) of larval aquatic invertebrates in artificial tree holes. Solid lines were calculated from the linear mixed effect models, one for abundance and one for species richness using abundance as a covariate and are shown for samplings in 2015. Gray bands show 95% confidence interval. Mean values and confidence interval are printed for June 2015 and June 2016 for the regions Alb (orange), Hainich (blue), and Schorfheide (green). Raw data are plotted as dots for the regions Alb (orange), Hainich (blue), and Schorfheide (green). Abundance and species richness were log‐transformed for the analysis, so log‐transformed values are also shown here. We used the package “ggbreak” (Xu et al. 2021) to introduce a gap in the x‐axis between the samplings in 2015 and 2016.

Within the first month, 80% of the artificial tree holes were colonized by invertebrates, and from the second month onwards, larval invertebrates were found in all containers. Abundance and species richness reached a maximum in July, followed by a decline in the following month and a subsequent increase in the following season, best captured by a third‐order polynomial function (Figure 1, Table 1 and Table S3). However, 15 months after deployment (June 2016) the mean values of overall abundance and region‐specific abundance were higher than in the comparable month of the previous year (Figure 1) and even exceeded the maximum values of the previous year, except in the region Schorfheide (Table S1).

Analysis of the interactions of plot‐ and tree hole‐scale variables with time showed that temporal dynamics in abundance and species richness were affected by water parameters (Table 1), that is, colonization was initially faster in oxygen‐rich water that supported higher abundances, and at near‐neutral pH values that favored greater species richness. Interestingly, this pattern was reversed in June 2016; we found more individuals in oxygen‐poor water and more species in alkaline water.

### 4.2 Effect of Forest Management and Dominant Tree Species on Abundance and Species Richness

**FIGURE 2:**
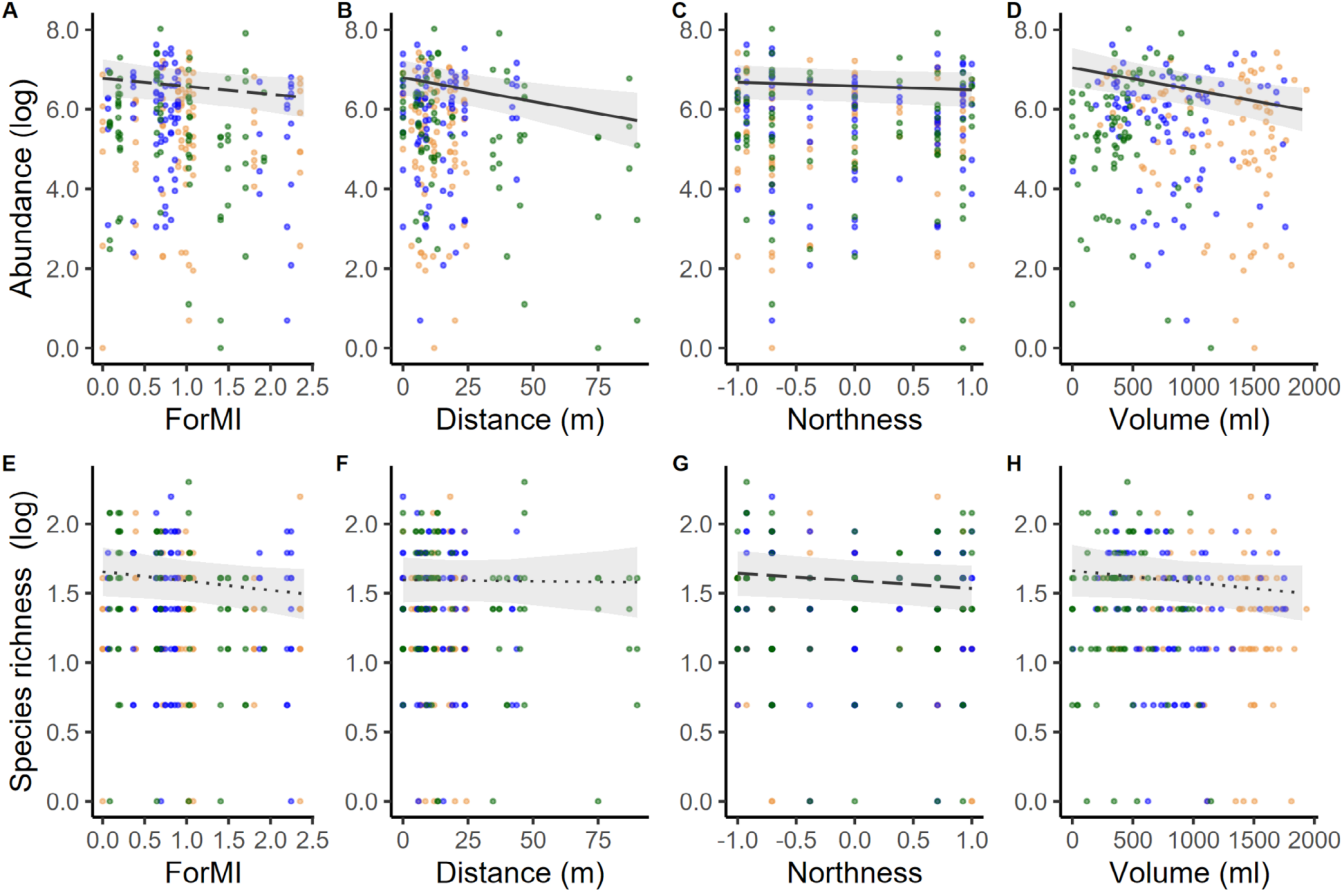
Effect of forest management intensity index (ForMI) (A,E), distance to the nearest natural tree hole (B,F), northness (C,G), water volume at the time of sampling (D,H) on total abundance and species richness in artificial tree holes in the regions Alb, Hainich, and Schorfheide based on the two separate linear mixed effect models, one for abundance and one for species richness using abundance as a covariate. Significant effects are printed as solid lines in B, C and D, marginal significant effects are printed as long dashed lines in A and G, and gray bands show 95% confidence interval. Raw data are plotted as dots for the regions Alb (orange), Hainich (blue), and Schorfheide (green). Abundance and species data were log‐transformed for the analysis, so log‐transformed values are also shown here.

Our results showed that forest management intensity (ForMI) had only a slight negative effect on insect larval abundance, while species richness remained unaffected (Table 1, Figure 2A,E). For the model without abundance as a covariate, a significant negative effect on species richness was observed (*F*_1,28_ = 4.37, *p* = 0.0459, Table S2). The dominant tree species of the plot had a strong significant effect on larval abundance: it was higher in artificial tree holes in plots dominated by beech trees (*F*_2,27_ = 10.01, *p* = 0.0006; Table S3 and Figure S3), but again, species richness was not affected when fitting abundance as a covariate, and it showed a significant response without using abundance as a covariate (*F*_2,27_ = 3.803, *p* = 0.0351). The distance to the nearest natural water‐filled tree hole had a strong negative effect on abundance. This effect was less significant in the model that included the dominant tree species of the stand (*F*_1,26_ = 4.220, *p* = 0.0501; Table S3). Species richness was not affected in either model (Table 1 and Figure 2B,F).

### 4.3 Effect of Microhabitat Properties on Abundance and Species Richness

Regarding microhabitat properties, total abundance of insect larvae in artificial tree holes was strongly negatively affected by water volume at the time of sampling. Northness had a weak effect on abundance with higher values in the south (Table 1 and Figure 2C,D). Species richness was not affected directly by any microhabitat property except northness (with the higher values in the south; Table 1, Table S3 and Figure S2G,H). For the model without abundance as a covariate, species richness was additionally affected by water volume (Table S2; F_1,169_ = 5.01, *p* = 0.0265). The type of debris inserted did not affect abundance or species richness. The two‐way interactions, neither between debris type and ForMI (Table 1), nor between debris type and dominant tree species, were significant (Table S3).

### 4.4 Community Composition

**FIGURE 3:**
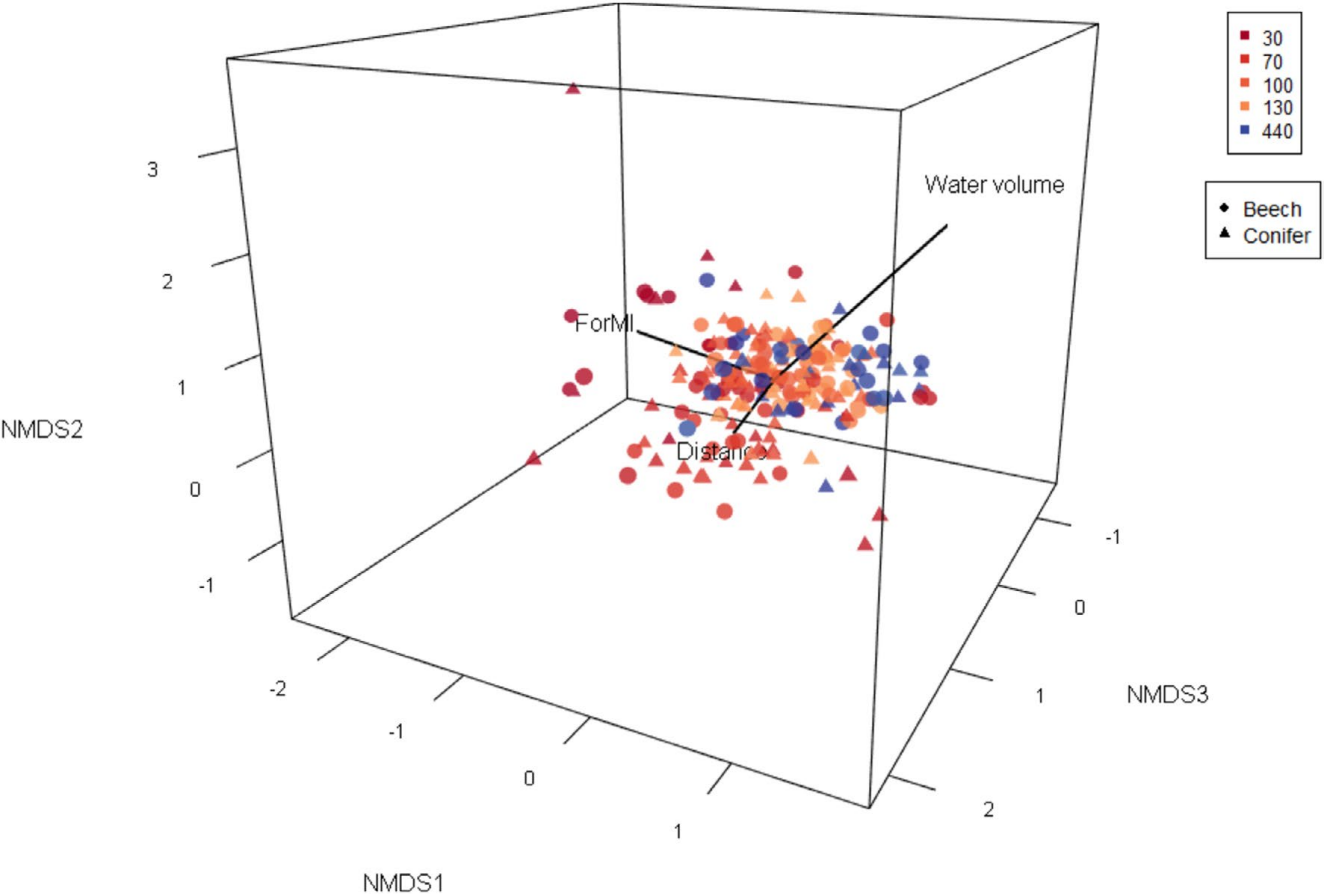
Three dimensional NMDS plot of artificial tree holes based on Bray–Curtis dissimilarity. Forest management intensity index (ForMI), distance to the nearest water filled tree holes, and water volume are shown as vectors calculated by using the *envfit* function from the package “vegan.” Symbol colors represent time is expressed as days since deployment of the containers. Symbols represent the different types of debris inserted into the container. Stress = 0.176.

PERMANOVA results showed that the community composition of artificial tree holes was significantly different between the study regions. Furthermore, community composition showed highly significant temporal dynamics (Table 1 and Figure 3). ForMI as well as the dominant tree species of the plot (*F*_2,236_ = 3.23, *p* = 0.001) and distance to the nearest water‐filled tree hole had a highly significant effect on community composition (Table 1, Figure 3, Table S3 and Figure S4). The only microhabitat property that affected community composition strongly was debris type; water volume at the time of sampling had only a weak effect (Table 1 and Figure 3). There was a significant effect of the two‐way interaction between debris type and ForMI (Table 1). Dissolved oxygen affected compositional changes over time (weak significant interaction effect; Table 1).

### 4.5 Most Abundant Species

**FIGURE 4:**
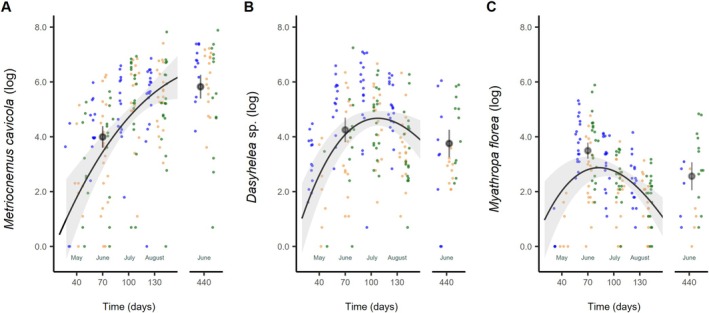
Temporal pattern of abundance of the three most abundant and frequent species *Metriocnemus cavicola* (A), *Dasyhelea* sp. 1 (B), and *Myathropa florea* (C). Raw data are plotted as dots for the regions Alb (orange), Hainich (blue), and Schorfheide (green). Solid lines were calculated from the linear mixed effect models and are shown for samplings in 2015. Mean values and confidence interval printed for June 2015 and 2016. *M. cavicola* followed a second order polynomial relationship. *Dasyhelea* sp. 1 and *M. florea* followed a third order polynomial relationship. Abundance data of the species are log‐transformed so log‐transformed values are also shown here.

The most abundant species in the artificial tree holes over the course of the study were the dipteran species *Metriocnemus cavicola* (Chironomidae, 61.6% of all collected individuals), followed by *Dasyhelea* sp. 1 (Ceratopogonidae, 28.7%) and *Myathropa florea* (Syrphidae, 4.8%) (Figures S5 and S6). These species were also the most frequently found species: *M. cavicola* colonized 253 containers (78.6%), *Dasyhelea* sp. 1 247 (76.7%), and *M. florea* 232 (72%). While the abundance of *M. cavicola* did not differ between regions, the abundances of *Dasyhelea* sp. 1 and *M. florea* were significantly higher in Hainich than in Alb and Schorfheide (Table 2, Table S1 and Figure S7). Time was a significant predictor of the abundance of the three most abundant species but affected them in different ways. *M. cavicola* increased in average abundance towards the end of the study period in 2015 and reached a maximum in the following year, best described by a second‐order polynomial relationship (Table 2, Figure 4A and Table S1). The abundances of *Dasyhelea* sp. 1 and *M. florea* were best described by a third‐order polynomial relationship, displaying a peak already in July and June 2015, respectively, followed by a decline in the following months and a subsequent increase in the following year. Fifteen months after deployment (June 2016) the mean values were generally lower than the mean values of the same month of the previous year, although there was large variation across regions (Figure 4B,C and Table S1).

**TABLE 2:** Results of the lme model testing the effect of explanatory variables and interactions on the abundance of the three most abundant and frequent species. Abundances of species were log‐transformed prior to the analysis. Individual tree ID nested in plot was specified as a random effect. Significant *p*‐values (< 0.05) are shown in bold, while 0.05 < *p* < 0.1 are shown in italic. Arrows indicate the positive (↑) or negative (↓) effects of numeric variables on response variables. For MI = Forest management intensity index. Estimated variances for the random effects: Plot 2.57e‐07, 5.45e‐08, 5.68e‐13; tree within plot 0.24, 0.19, 1.75e‐08.

	ndf	*Metriocnemus cavicola*				*Dasyhelea* sp. 1				*Myathropa florea*			
		ddf	*F*	*R* ^2^	*p*	ddf	*F*‐value	*R* ^2^	*p*	ddf	*F*‐value	*R* ^2^	*p*
Region	2	28	1.36	0.037	0.2732	28	7.71	0.147	**0.0021**	28	13.58	0.051	**0.0001**
ForMI	1	28	9.26	0.010	**0.0050** ↓	28	0.10	0.010	0.7571	28	0.10	0.005	0.7537
Distance to nearest natural tree hole (m)	1	27	3.56	0.005	*0.0700*	27	4.55	0.013	**0.0423** ↓	27	0.16	0.002	0.6950
Debris type	1	27	0.73	0.002	0.4014	27	0.72	0.003	0.4027	27	1.60	0.005	0.2172
Time (days since set‐up)	1	135	44.39	0.064	**< 0.0001**	123	4.47	0.221	**0.0365**	114	3.14	0.134	*0.0793*
Time (second order polynomial)	1	135	46.94	0.019	**< 0.0001**	123	14.61	0.160	**0.0002**	114	12.11	0.190	**0.0007**
Time (third order polynomial)	1	135	1.65	0.008	0.2009	123	42.37	0.140	**< 0.0001**	114	32.09	0.186	**< 0.0001**
Northness		135	0.91	0.002	0.3407	123	0.01	0.005	0.9391	114	1.36	0.021	0.2461
Water volume (ml)	1	135	0.23	0.006	0.6306	123	9.81	0.026	**0.0022** ↓	114	17.90	0.049	**0.0001** ↓
Disolved oxygen (mg/l)	1	135	1.31	0.001	0.2543	123	1.10	0.041	0.3060	114	0.80	0.030	0.3742
pH	1	135	0.71	0.008	0.4015	123	0.03	0.000	0.9581	114	0.28	0.003	0.8686
ForMI:Debris type	1	27	0.01	0.000	0.9168	27	0.41	0.000	0.5271	27	0.50	0.002	0.4853
ForMI:Time	1	135	0.01	0.001	0.9123	123	0.83	0.007	0.3652	114	0.56	0.008	0.4547
Distance to nearest natural tree hole:Time	1	135	0.15	0.000	0.7034	123	0.70	0.000	0.4048	114	0.28	0.002	0.5958
Debris type:Time	1	135	0.18	0.000	0.6724	123	0.14	0.001	0.7103	114	0.31	0.001	0.5749
Water volume:Time	1	135	1.39	0.007	0.2413	123	0.02	0.000	0.78905	114	0.02	0.000	0.8888
Dissolved oxygen:Time	1	135	3.35	0.008	*0.0693*	123	5.42	0.043	**0.0216**	114	3.39	0.034	*0.0684*
pH:Time	1	135	0.80	0.003	0.3733	123	0.38	0.005	0.5372	114	0.65	0.025	0.4202

**FIGURE 5:**
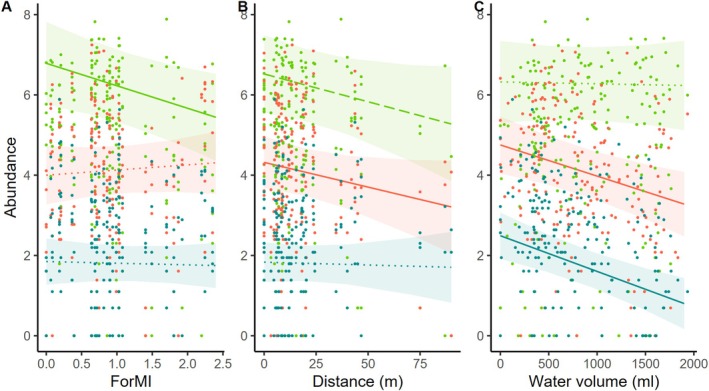
Effect plots of explanatory variables forest management intensity index (ForMI; A), distance to nearest natural tree hole (B), and water volume (C) at the time of sampling on the abundance of *Metriocnemus cavicola* (green), *Dasyhelea* sp. 1 (orange) and *Myathropa florea* (blue) in artificial tree holes based on linear mixed effect models. Only significant explanatory variables are shown (solid lines, see Table 2). Colored bands show 95% confidence interval. Raw data are plotted as dots for the regions Alb (orange), Hainich (blue), and Schorfheide (green). Abundance data of the species are log‐transformed so log‐transformed values are also shown here. Distance = distance to the nearest natural water‐filled tree hole.

We found no statistically significant two‐way interaction effects of plot‐ and tree hole‐scale variables with time on the abundances of these species, except for the two‐way interaction of dissolved oxygen and time (Table 2). Using the model with dominant tree species instead of ForMI, we found similar results (Table S4). Of the three most abundant species, only *M. cavicola* responded negatively to an increase in ForMI (Table 2 and Figure 5A) and was significantly more abundant in plots dominated by beech trees (*F*_2,27_ = 10.28, *p* = 0.0005; Table S4). Increasing distance to the nearest natural water‐filled tree hole had a significant negative effect only on the abundance of *Dasyhelea* sp. 1 (Table 2 and Figure 5B). The three most abundant species responded differently to microhabitat properties: High water volume at the time of sampling had a strong negative effect on the abundance of *Dasyhelea* sp. 1 and *M. florea* (Table 2 and Figure 5C).

Neither the type of debris inserted nor any two‐way interaction, that is, with ForMI (Table 2), or dominant tree species, respectively affected the abundance of these species (Table S4).

## 5 Discussion

Our results show a dynamic temporal pattern of colonization of new microhabitats by larval aquatic insects. Forest management practices primarily influenced overall insect abundance and community composition by altering tree species composition. Additionally, there was a strong effect of forest management intensity on community composition. Among the microhabitat properties, debris type significantly influenced community composition. Furthermore, the distance to the nearest natural tree hole and water volume had strong species‐specific impacts on larval abundance.

### 5.1 Temporal Patterns

Our first aim was to investigate the colonization patterns of artificial water‐filled tree holes by larval aquatic insect communities, and the results revealed rapid colonization within 2 months after deployment of the container. This supports the results of a previous study that found artificial tree holes to be colonized by insect larvae, that is, *Dasyhelea* sp., after 2 months (Ptatscheck and Traunspurger 2014). In addition, we observed a seasonal peak in abundance and an even higher number of individuals in the second year, which is in line with our first hypothesis. Species richness increased in the first 3 months but, in contrast to abundance, it did not increase further in the second year. Similar to our study, a seasonality‐dependent occurrence of macrofaunal taxa and temporal changes in community composition were previously observed in both artificial (Harlan and Paradise 2006; Paradise et al. 2008; Ptatscheck and Traunspurger 2015) and natural (Devetter 2004) tree holes. In contrast, another long‐term study on insect larvae in natural tree holes showed a seasonally independent periodicity of insect communities, mainly due to irregular drying events (Gossner 2018) that we excluded by refilling the containers regularly. The observed changes in community composition suggest that forming complete communities takes time and is likely influenced by oviposition decisions, order of arrival, and life cycle duration. The increased insect abundance in the second year may be due to additional colonization of species with limited dispersal abilities that require longer to arrive and establish in a new habitat. Furthermore, species with specific traits that provide a competitive advantage, such as superior frost resistance, effective overwintering strategies, or short generation times, may be particularly effective in colonizing new habitats. In addition, individual species might benefit from higher nutrient availability through decomposed debris or additional input of leaf litter entering despite the roofs more than others. We suppose that the changes in community composition observed in our study may be attributed to the different colonization patterns of the most abundant and frequent species, the chironomid *Metriocnemus cavicola*, the ceratopogonid *Dasyhelea* sp. 1, and the syrphid *Myathropa florea*, with *M. cavicola* increasing continuously *and Dasyhelea* sp. 1 and *M. florea* larvae displaying seasonal peaks. In the second year, the increase in *M. cavicola* abundance might explain the higher total numbers of individuals, suggesting superior overwintering strategies for *M. cavicola* and ceratopogonid species (Harlan and Paradise 2006; Gossner 2018) enabling them to keep their populations at a high level over the winter months. However, we cannot rule out the possibility that different dynamics would have emerged if the experiment had lasted longer. We suppose that temporal dynamics are probably driven by dispersal and competitive abilities as well as by different life‐history traits such as generation time and resistance and resilience to extreme events such as drought and freezing. This calls for long‐term studies of insect community dynamics that integrate species traits and the fluctuation of abiotic conditions to better understand the temporal dynamics of these communities.

### 5.2 Effects of Forest Management

Our second aim focused on the effects of parameters related to forest management. The observed strong effects of forest management intensity, in terms of ForMI, on insect community composition confirm the results of previous studies conducted in the same regions (Gossner et al. 2016; Petermann et al. 2016). However, we found only a weak, negative effect of increasing ForMI on overall larval abundance, differing from the stronger effects reported in these previous studies. This discrepancy may be attributed to differences in methodological settings, including the number of sampling events, with our study covering an entire season compared to the one or two discrete sampling events of these studies. By testing the effects of the dominant tree species of the stand, which also express management decisions, we showed changes in community composition and significantly lower abundances in forests dominated by conifers than in forests dominated by beech trees, confirming our hypothesis. Our results emphasize the importance of tree species composition in shaping the community structure of insect larvae inhabiting tree holes, likely by both altering microhabitat availability and microhabitat properties (Gossner et al. 2016; Petermann et al. 2020, 2016). In our study, we used the distance to the nearest natural tree hole, which was previously shown to be significantly affected by forest management (Gossner et al. 2016), as a proxy for microhabitat availability. As expected, a significant increase in this distance led to a shift in community composition as well as a significant decrease in the total abundance of insect larvae. Previous studies provided inconsistent results in this respect (Gossner et al. 2016; Petermann et al. 2020, 2016) warranting further research into this parameter and potential dispersal limitation in this system in general. In conclusion, our results demonstrate that colonization patterns were strongly influenced by forest management. However, this influence is only partially captured by the forest management index (FORMI). While insect abundances appeared to be only marginally affected, community composition showed a strong response throughout the experiment. This is likely due to altered tree composition and associated increasing dispersal distances between microhabitats, which in turn affected both abundance and community composition through altered colonization patterns. Overall, the results provide substantial support for our hypothesis.

We found different responses of individual species to forest management‐related parameters. While *M. cavicola* was strongly negatively affected by increasing ForMI and was less abundant per tree hole in forests dominated by conifers, *Dasyhelea* sp. 1 and *M. florea* were not affected by forest management‐related parameters at all. Species‐specific dispersal abilities may influence colonization patterns: strong flyers among the Eristalinae, such as *Eristalis tenax* and presumably also *M. florea* (Pruner 2016; Thyselius et al. 2023) may have an advantage early in the season and in plots dominated by conifers with fewer habitats and thus greater dispersal distances. Conversely, *Dasyhelea* sp., as a likely weaker flier, and *M. cavicola* may dominate in beech forests due to possible competitive advantages such as high fecundity, short developmental times, or tolerance to harsh conditions. A recent study including functional traits of common tree hole inhabiting insects (Petermann et al. 2020) found that species respond to changes in habitat availability caused by forest management on the basis of specific traits such as adult wing length and particular overwintering strategies. This again points to the need for trait databases with detailed information on life history, tolerance to harsh abiotic conditions, colonization, and competitive abilities to support the prediction of management impacts on individual species and communities.

### 5.3 Effects of Microhabitat Properties

Our third aim was to test the effects of microhabitat properties on tree hole inhabiting larval insects, and we identified several effects. First, the type of debris inserted into the container as resources of different quality shaped the composition of insect communities throughout the study period. Moreover, the observed interaction effect indicates that this influence is strongly modulated by the intensity of forest management. According to previous studies (Gossner et al. 2016; Petermann et al. 2016) forest management indirectly influences community composition through changes in debris amount and associated water parameters. Although we did not quantify additional debris input and decomposition in our study, our results indicate that debris quality influences community composition. This supports previous studies mostly conducted in other types of aquatic ecosystems, showing differences in insect colonization by insects between habitats with deciduous litter and those with coniferous litter (e. g. Hisabae et al. 2011; Pintar and Resetarits 2017; Rosset and Bärlocher 1982; Sedell et al. 1975; Yanoviak 1999; Yee and Juliano 2006). As shown for beetles in mesocosms (Pintar and Resetarits 2017) preferences for certain food sources, specifically debris types that differ in accessibility and nutrient content, may have similarly influenced the community composition of tree hole inhabiting insects in our study. Further, water chemistry is an important factor and is likely influenced to a large extent by the different debris types as they vary in their chemical composition (Yanoviak 1999). The breakdown of leaf debris and other organic matter and biochemical changes due to microbes are reported to be important in changing water chemistry (Paradise and Dunson 1997; Walker et al. 1991). In our study, dissolved oxygen and pH did not directly affect any of our response variables, but we found changes in the effects of oxygen and pH on abundance, species richness, and community composition over time. Thus, we suppose that decomposition processes may have indirectly shaped insect communities by gradually modifying chemical water parameters over time, supporting our hypothesis. Second, the microhabitat properties, water volume, and northness played a significant role. Water volume, as a measure for tree‐hole size, had a strong negative impact on larval insect abundance persisting throughout our experiment, supporting our hypothesis, but it was of minor importance for community composition, most likely due to refilling. In contrast, results of previous studies on the effect of water volume on larval insect densities and species richness showed predominantly positive effects in natural as well as artificial tree holes (e. g. Gossner et al. 2016; Paradise 2004; Paradise et al. 2008; Sota 1996; Yoshida et al. 2018). Changes in community composition have been shown to be influenced by habitat size in general (Sota 1998) and by decreasing water volume during periods of drought in particular (Gossner 2018). Northness was used as a proxy for sun exposure, subsequently influencing water temperature, evaporation, and water volume. This parameter was associated with higher abundance and species richness in the south, where temperature is presumably higher and water volume was lower. Warmer water accelerates the development and growth rates of many aquatic insect larvae (Del‐Claro and Guillermo 2019; Lopez et al. 2019) and influences oviposition timing of mosquitos (see Day 2016), potentially leading to higher abundances. We also observed species‐specific responses to microhabitat properties: *M. cavicola* was more numerous in warmer water and tolerated the low oxygen levels in the second year of the experiment (see also Gossner 2018; Gossner and Petermann 2022). *Dasyhelea* sp.1 prospered at lower water volumes (see also Green 1990; Kitching 1971) which suggests a competitive advantage due to their fast development and higher tolerance to low water levels (Gossner and Petermann 2022) and *M. florea* preferred less humid habitats and lower temperatures (see also Ptatscheck and Traunspurger 2015).

## 6 Conclusion

Our findings emphasize the key role of tree‐related microhabitats for insect biodiversity in forests and the complex consequences of intensive forest management for the associated communities (Gossner et al. 2016; Petermann et al. 2016; Shipley et al. 2023). Our study shows complex and partly species‐specific patterns of colonization of artificial water‐filled tree holes. We found strong negative effects of forest management intensity and of the dominant tree species and dispersal distances on tree‐hole inhabiting insects throughout the colonization process. Our findings further suggest that microhabitat properties such as dispersal distances, debris type, and chemical water parameters of the tree holes strongly shape tree‐hole communities. We recommend that in addition to designating natural forest reserves and old‐growth forests, forest management plans should include the preservation and promotion of temporary water bodies such as water‐filled tree holes. This can be done by promoting mixed forest stands with tree species that develop water‐filled tree holes at an earlier age (e. g. beech) and by incorporating veteran and habitat trees with their water‐accumulating structures (Bütler et al. 2013; Horák 2017; Shipley et al. 2023). In addition, maintaining or creating stepping stones, that is, small habitat patches that act as connectors and allow insects to access distant habitat patches for colonization, can facilitate genetic exchange and contribute to the long‐term preservation of populations (Lapin et al. 2024).

Currently, knowledge regarding specific requirements of aquatic insects in water‐filled tree holes is limited. Our study provides valuable insights into the colonization patterns of these insects, both at the community and species level; it additionally highlights the suitability of artificial tree holes as a simple indicator system that enables rapid detection and quantification of changes in response to forest management and other anthropogenic impacts (Petermann et al. 2016). Future research should focus on species‐specific traits of adult and larval insects, such as those related to dispersal, feeding, competitive, and overwintering strategies, to enhance our understanding of these processes to facilitate the conservation of water‐filled tree holes and their inhabiting communities.

## Author Contributions

**Heidi Bartel:** data curation (equal), formal analysis (lead), investigation (supporting), validation (equal), visualization (lead), writing – original draft (lead). **Martin M. Gossner:** conceptualization (supporting), funding acquisition (supporting), investigation (supporting), methodology (equal), project administration (supporting), resources (supporting), supervision (supporting), writing – original draft (supporting). **Jana S. Petermann:** conceptualization (lead), data curation (equal), formal analysis (supporting), funding acquisition (lead), investigation (lead), methodology (equal), project administration (lead), resources (lead), supervision (lead), validation (equal), visualization (supporting), writing – original draft (supporting).

## Conflicts of Interest

The authors declare no conflicts of interest.

## Supporting information


**Data S1:** ece371962‐sup‐0001‐Supinfo01.docx.

## Data Availability

Data are available at https://datadryad.org/dataset/doi:10.5061/dryad.1ns1rn964.
