# Supplementary material for: Forest Management and the Colonization of Artificial Tree Holes by Aquatic Insect Larvae
Source: Ecol Evol. 2025 Aug 15;15(8):e71962. doi: 10.1002/ece3.71962 (PMC12356645; doi:10.1002/ece3.71962)
Supplement: Supplementary file 1 — Data S1: ece371962‐sup‐0001‐Supinfo01.docx. [file ECE3-15-e71962-s001.docx]

Supporting information Table. 1. Abundance, species richness, and abundance of the most abundant and frequent species per artificial tree holes, in all regions plots, and in the regions Schwäbische Alb (Alb), Hainich-Dün (Hainich), and Schorfheide-Chorin (Schorfheide), at the five sampling time points. Means ± standard errors (SE) are shown.

|  | May 2015 | | June 2015 | | July 2015 | | August 2015 | | June 2016 | |
| --- | --- | --- | --- | --- | --- | --- | --- | --- | --- | --- |
| *Abundance* | mean | SE | mean | SE | mean | SE | mean | SE | mean | SE |
| All plots | 30 | 6 | 304 | 34 | 697 | 77 | 498 | 54 | 795 | 88 |
| Alb | 23 | 10 | 219 | 36 | 532 | 85 | 433 | 78 | 680 | 122 |
| Hainich | 39 | 9 | 382 | 58 | 735 | 104 | 547 | 52 | 1058 | 163 |
| Schorfheide | 28 | 11 | 323 | 76 | 835 | 184 | 518 | 135 | 726 | 168 |
| *Species richness* |  |  |  |  |  |  |  |  |  |  |
| All plots | 1.8 | 0.2 | 4.6 | 0.2 | 5.3 | 0.2 | 4.4 | 0.2 | 4.8 | 0.3 |
| Alb | 1.9 | 0.3 | 4.5 | 0.3 | 5.1 | 0.4 | 4.2 | 0.2 | 4.5 | 0.4 |
| Hainich | 2.2 | 0.3 | 4.8 | 0.3 | 5.2 | 0.3 | 4.3 | 0.3 | 4.8 | 0.5 |
| Schorfheide | 1.2 | 0.2 | 4.4 | 0.2 | 5.7 | 0.4 | 4.9 | 0.3 | 5.3 | 0.5 |
| *Metriocnemus* |  |  |  |  |  |  |  |  |  |  |
| All plots | 14.1 | 4.7 | 75.0 | 14.1 | 341.7 | 51.1 | 355.2 | 49.1 | 671.4 | 88.0 |
| Alb | 12.6 | 7.9 | 97.7 | 31.6 | 350.6 | 87.1 | 352.0 | 79.3 | 630.0 | 117.0 |
| Hainich | 6.1 | 4.5 | 71.7 | 21.7 | 217.9 | 48.6 | 322.9 | 53.2 | 873.6 | 172.9 |
| Schorfheide | 23.5 | 10.6 | 54.5 | 16.4 | 445.4 | 110.0 | 389.3 | 113.3 | 560.2 | 172.1 |
| *Dasyhelea* |  |  |  |  |  |  |  |  |  |  |
| All plots | 9.1 | 2.3 | 123.8 | 27.0 | 305.0 | 52.3 | 123.3 | 21.1 | 69.7 | 16.5 |
| Alb | 6.4 | 2.7 | 84.0 | 30.4 | 140.8 | 35.5 | 68.0 | 23.9 | 9.5 | 2.5 |
| Hainich | 20.1 | 5.7 | 178.5 | 43.3 | 461.8 | 86.8 | 196.0 | 36.4 | 99.2 | 42.6 |
| Schorfheide | 1.5 | 1.5 | 115.4 | 61.6 | 333.3 | 120.9 | 111.4 | 43.8 | 110.3 | 28.4 |
| *Myathropa* |  |  |  |  |  |  |  |  |  |  |
| All plots | 0.4 | 0.2 | 59.8 | 10.0 | 21.8 | 2.5 | 8.8 | 1.2 | 14.3 | 3.9 |
| Alb | 0.9 | 0.4 | 24.0 | 5.8 | 13.9 | 2.4 | 5.6 | 2.4 | 1.4 | 0.9 |
| Hainich | 0.3 | 0.2 | 68.1 | 12.0 | 30.6 | 4.4 | 12.5 | 4.4 | 6.1 | 2.5 |
| Schorfheide | 0.0 | 0.0 | 89.7 | 25.2 | 22.1 | 5.2 | 8.5 | 5.2 | 33.9 | 9.0 |

Supporting information Table 2. Results of the lme models testing the effect of explanatory variables (including ForMI) and interactions on species richness (log-transformed) excluding abundance as a covariate. Individual tree number was specified as a random effect nested in plot. Significant p-vales (<0.05) are shown in bold, while 0.05<P<0.1 are shown in italic. ForMI = Forest management intensity index.

|  |  | *Species richness* | | |
| --- | --- | --- | --- | --- |
|  | ndf | ddf | F-value | p-value |
| Region | 2 | 28 | 1.55 | 0.2291 |
| ForMI | 1 | 28 | 4.37 | **0.0459** ⭣ |
| Distance to nearest natural tree hole (m) | 1 | 27 | 2.13 | 0.1556 |
| Debris type | 1 | 27 | 0.22 | 0.6405 |
| Time (days since set-up) | 1 | 175 | 26.00 | **<.0001** |
| Time (second order polynomial) | 1 | 175 | 56.75 | **<.0001** |
| Time (third order polynomial) | 1 | 175 | 36.91 | **<.0001** |
| Northness | 3 | 175 | 5.86 | **0.0165** |
| Water volume (ml) | 1 | 175 | 5.01 | **0.0265** ⭣ |
| Dissolved oxygen (mg/l) | 1 | 175 | 0.50 | 0.4891 |
| pH | 1 | 175 | 0.01 | 0.9104 |
| ForMI:Debris type | 1 | 27 | 0.47 | 0.4987 |
| ForMI:Time | 1 | 175 | 1.83 | 0.1775 |
| Distance to nearest natural tree hole:Time | 1 | 175 | 1.74 | 0.1889 |
| Debris type:Time | 1 | 175 | 2.07 | 0.1516 |
| Water volume:Time | 1 | 175 | 0.17 | 0.6797 |
| Dissolved oxygen:Time | 1 | 175 | 0.71 | 0.4008 |
| pH:Time | 1 | 175 | 10.39 | **0.0015** |

Supporting information Table 3. Results of the lme model (abundance, species richness) and PERMANOVA (community composition) testing the effect of explanatory variables (dominant tree species of the stand instead of ForMI) and interactions on abundance, species richness and composition of the communities. Abundance and species richness were log-transformed prior to the analysis. Abundance (log-transformed) is used as a covariate in the analysis of species richness. In the lme models individual tree number was specified as a random effect nested in plot. In the PERMANOVA plot was specified as blocking factor for permutation tests using the blocks argument. Significant p-vales (<0.05) are shown in bold, while 0.05<P<0.1 are shown in italic. Estimated variances for the random effects: plot 2.96e-15, 3.91e-13, tree within plot 4.15e-08, 9.5e-10.

|  |  | *Abundance* | | | | *Species richness* | | | | *Community composition* | | |
| --- | --- | --- | --- | --- | --- | --- | --- | --- | --- | --- | --- | --- |
|  | ndf | ddf | F-value | p-value | R^2^ | ddf | F-value | p-value | R^2^ | F-value | R^2^ | p-value |
| Abundance | 1 |  |  |  |  | 167 | 146.98 | **<.0001** | 0.060 |  |  |  |
| Region | 2 | 27 | 3.12 | *0.0604* | 0.161 | 27 | 1.00 | 0.3823 | 0.022 | 3.73 | 0.025 | **0.0001** |
| Dominant tree species | 2 | 27 | 10.01 | **0.0006** | 0.040 | 27 | 0.28 | 0.7987 | 0.012 | 3.23 | 0.022 | **0.0001** |
| Distance to nearest natural tree hole (m) | 1 | 26 | 4.22 | *0.0501* | 0.000 | 26 | 0.20 | 0.6676 | 0.005 | 1.32 | 0.004 | **0.0001** |
| Debris type | 1 | 26 | 1.46 | 0.2373 | 0.011 | 26 | 0.02 | 0.8838 | 0.001 | 1.22 | 0.004 | **0.0001** |
| Time (days since set-up) | 1 | 174 | 93.72 | **<.0001** | 0.271 | 173 | 0.36 | 0.5483 | 0.067 | 16.62 | 0.056 | **0.0001** |
| Time (second order polynomial) | 1 | 174 | 164.95 | **<.0001** | 0.165 | 173 | 4.61 | **0.0332** | 0.072 | 20.72 | 0.069 | **0.0001** |
| Time (third order polynomial) | 1 | 174 | 48.77 | **<.0001** | 0.109 | 173 | 14.79 | **0.0002** | 0.059 | 10.87 | 0.037 | **0.0001** |
| Northness | 1 | 174 | 5.16 | **0.0241** | 0.009 | 173 | 3.75 | *0.0544* | 0.011 | 1.35 | 0.014 | 0.2124 |
| Water volume (ml) | 1 | 174 | 11.06 | **0.0011** | 0.048 | 173 | 0.80 | 0.3736 | 0.000 | 2.31 | 0.007 | *0.0815* |
| Dissolved oxygen (mg/l) | 1 | 174 | 1.80 | 0.1814 | 0.043 | 173 | 0.80 | 0.3706 | 0.005 | 1.30 | 0.004 | 0.2454 |
| pH | 1 | 174 | 1.63 | 0.2041 | 0.039 | 173 | 0.08 | 0.7899 | 0.021 | 0.44 | 0.002 | 0.8025 |
| Dominant tree species:Debris type | 2 | 26 | 0.72 | 0.4949 | 0.009 | 26 | 1.49 | 0.2442 | 0.009 | 0.72 | 0.005 | 0.0477 |
| Dominant tree species:Time | 2 | 174 | 0.24 | 0.7900 | 0.007 | 173 | 0.90 | 0.4089 | 0.006 | 1.07 | 0.007 | 0.3558 |
| Distance to nearest natural tree hole:Time | 1 | 174 | 0.18 | 0.7117 | 0.000 | 173 | 4.90 | 0.0499 | 0.012 | 1.07 | 0.004 | 0.2998 |
| Debris type:Time | 1 | 174 | 0.27 | 0.6026 | 0.000 | 173 | 2.55 | 0.1122 | 0.006 | 0.69 | 0.002 | 0.6130 |
| Water volume:Time | 2 | 174 | 2.86 | *0.0928* | 0.014 | 173 | 0.67 | 0.4140 | 0.002 | 1.10 | 0.004 | 0.2832 |
| Dissolved oxygen:Time | 1 | 174 | 14.89 | **0.0002** | 0.029 | 173 | 0.04 | 0.8339 | 0.004 | 2.39 | 0.007 | **0.0470** |
| pH:Time | 1 | 174 | 5.69 | **0.0182** | 0.021 | 173 | 6.97 | **0.0091** | 0.026 | 0.41 | 0.002 | 0.8817 |

Supporting information Table 4. Results of the lme models testing the effect of explanatory variables including dominant tree species of the stand as predictor variable and interactions on the abundance of the three most abundant and frequent species. Abundances of species were log-transformed prior to the analysis. Individual tree ID was specified as a random effect nested in plot. 0.05<P<0.1 are printed in bold and italic, respectively.

|  | ndf | *Metriocnemus cavicola* | | | *Dasyhelea* sp. 1 | | | *Myathropa florea* | | |
| --- | --- | --- | --- | --- | --- | --- | --- | --- | --- | --- |
|  |  | ddf | F-value | p-value | ddf | F-value | p-value | ddf | F-value | p-value |
| Region | 2 | 27 | 1.43 | 0.2566 | 27 | 7.63 | **0.0024** | 27 | 13.64 | **0.0001** |
| Dominant tree species | 2 | 27 | 10.28 | **0.0005** | 27 | 1.13 | 0.3386 | 27 | 2.23 | 0.1274 |
| Distance to nearest natural tree hole (m) | 1 | 26 | 0.04 | 0.8372 | 26 | 1.49 | 0.2339 | 26 | 0.89 | 0.3544 |
| Debris type | 1 | 26 | 0.63 | 0.4354 | 26 | 0.53 | 0.4748 | 26 | 1.96 | 0.1731 |
| Time (days since set-up) | 1 | 134 | 45.56 | **<.0001** | 122 | 4.31 | **0.0399** | 113 | 3.79 | *0.0539* |
| Time (second order polynomial) | 1 | 134 | 48.49 | **<.0001** | 122 | 14.47 | **0.0002** | 113 | 11.98 | **0.0008** |
| Time (third order polynomial) | 1 | 134 | 1.98 | 0.1616 | 122 | 41.88 | **<.0001** | 113 | 31.06 | **<.0001** |
| Cardinal direction | 3 | 134 | 1.37 | 0.2445 | 122 | 0.0001 | 0.9923 | 113 | 0.91 | 0.3422 |
| Water volume (ml) | 1 | 134 | 0.0000 | 0.9993 | 122 | 9.99 | **0.0020** ⭣ | 113 | 15.88 | **0.0001** ⭣ |
| Dissolved oxygen (mg/l) | 1 | 134 | 0.57 | 0.4533 | 122 | 1.44 | 0.2332 | 113 | 1.34 | 0.2487 |
| pH | 1 | 134 | 1.40 | 0.2384 | 122 | 0.0005 | 0.9823 | 113 | 0.07 | 0.7889 |
| Dominant tree species:Debris type | 2 | 26 | 0.38 | 0.6863 | 26 | 0.99 | 0.3844 | 26 | 0.01 | 0.9866 |
| Dominant tree species:Time | 2 | 134 | 0.41 | 0.6634 | 122 | 0.07 | 0.9307 | 113 | 0.53 | 0.5873 |
| Distance to nearest natural tree hole:Time | 1 | 134 | 0.56 | 0.4566 | 122 | 0.68 | 0.4098 | 113 | 0.10 | 0.7487 |
| Debris type:Time | 1 | 134 | 0.82 | 0.3658 | 122 | 0.15 | 0.6978 | 113 | 0.05 | 0.8164 |
| Water volume:Time | 2 | 134 | 1.11 | 0.2940 | 122 | 0.03 | 0.8645 | 113 | 0.01 | 0.9030 |
| Dissolved oxygen:Time | 1 | 134 | 4.02 | **0.0472** | 122 | 4.96 | **0.0279** | 113 | 4.55 | **0.0352** |
| pH:Time | 1 | 134 | 3.59 | *0.0602* | 122 | 0.65 | 0.4202 | 113 | 0.92 | 0.3392 |

Supporting information Fig. 1. View of the experimental setup on a spruce (*Picea abies*) tree showing the construction and positioning of containers used as analogues to natural tree holes with mesh roofs to keep out additional detritus.


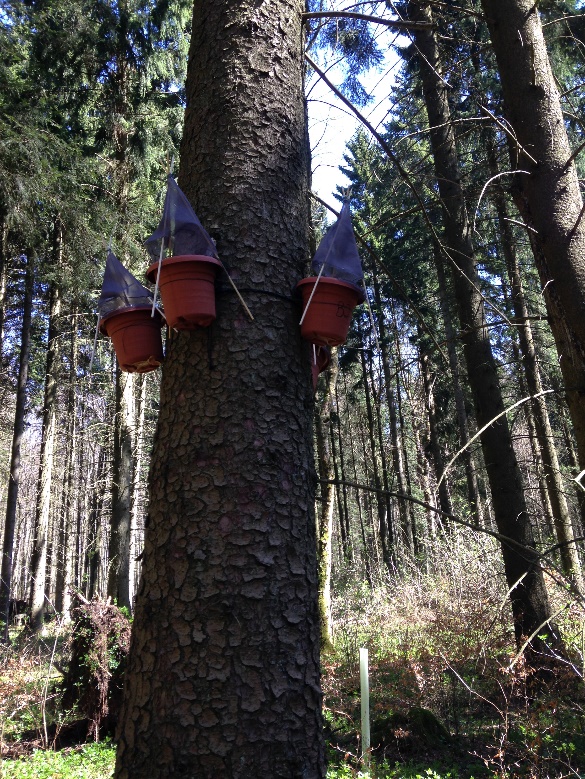


Supporting information Fig. 2. Correlation matrix (Spearman correlation coefficient) of explanatory variables for the artificial tree hole data set. Distance = distance to the nearest natural tree hole (m), ForMI = forest management intensity index, Time = days since setup.


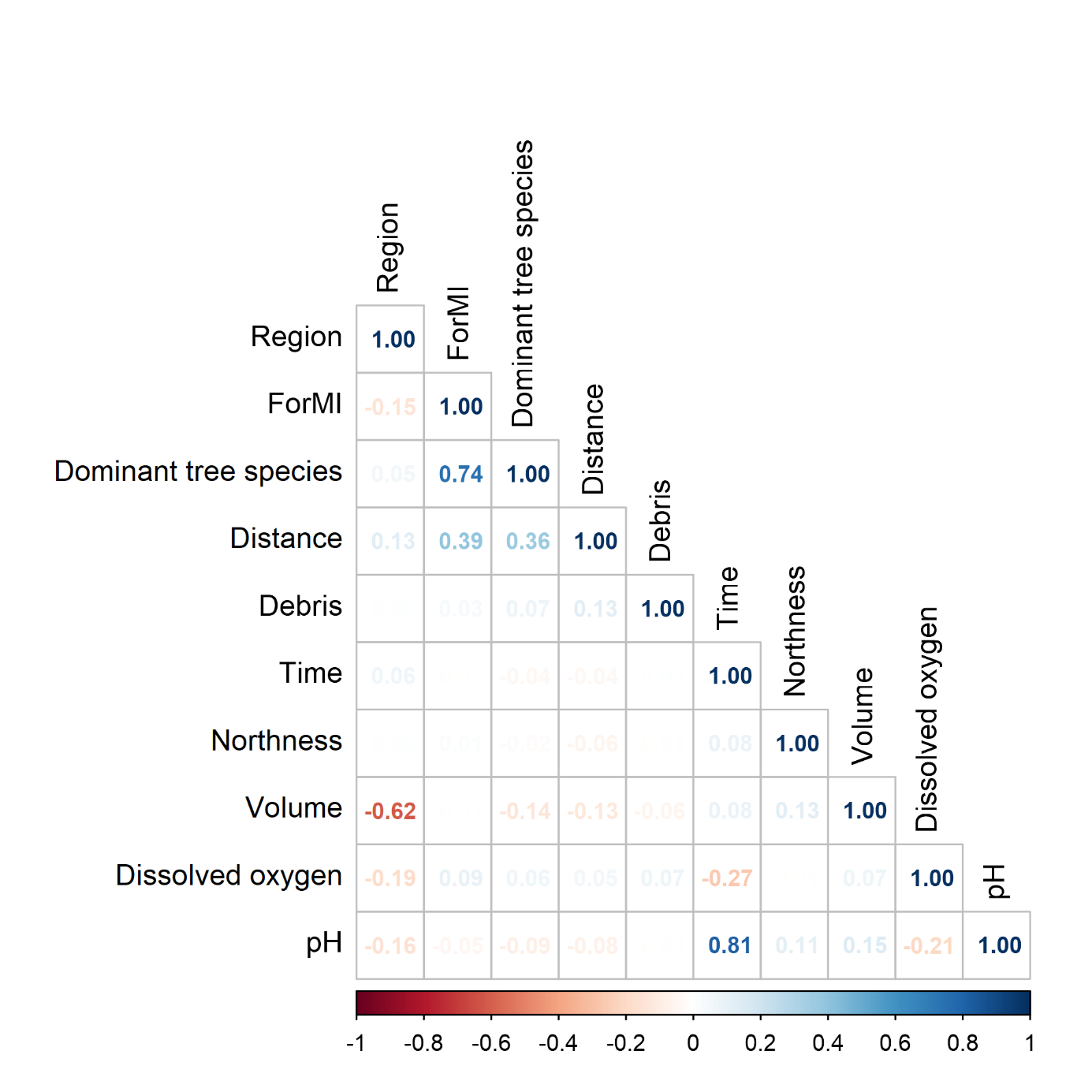


Supporting information Fig.3. Effect of dominant tree species of the stand on total abundance (significant; A) and species richness (non-significant; B) based on the linear mixed effect models (see Supporting information Table 3) are printed as black dots and lines showing 95% confidence interval. Mean values and confidence interval are printed for the regions Alb (orange), Hainich (blue), and Schorfheide (green). Raw data are plotted as dots for the regions Alb (orange), Hainich (blue), and Schorfheide (green). Abundance and species data were log-transformed for the analysis, so log-transformed values are also shown here.


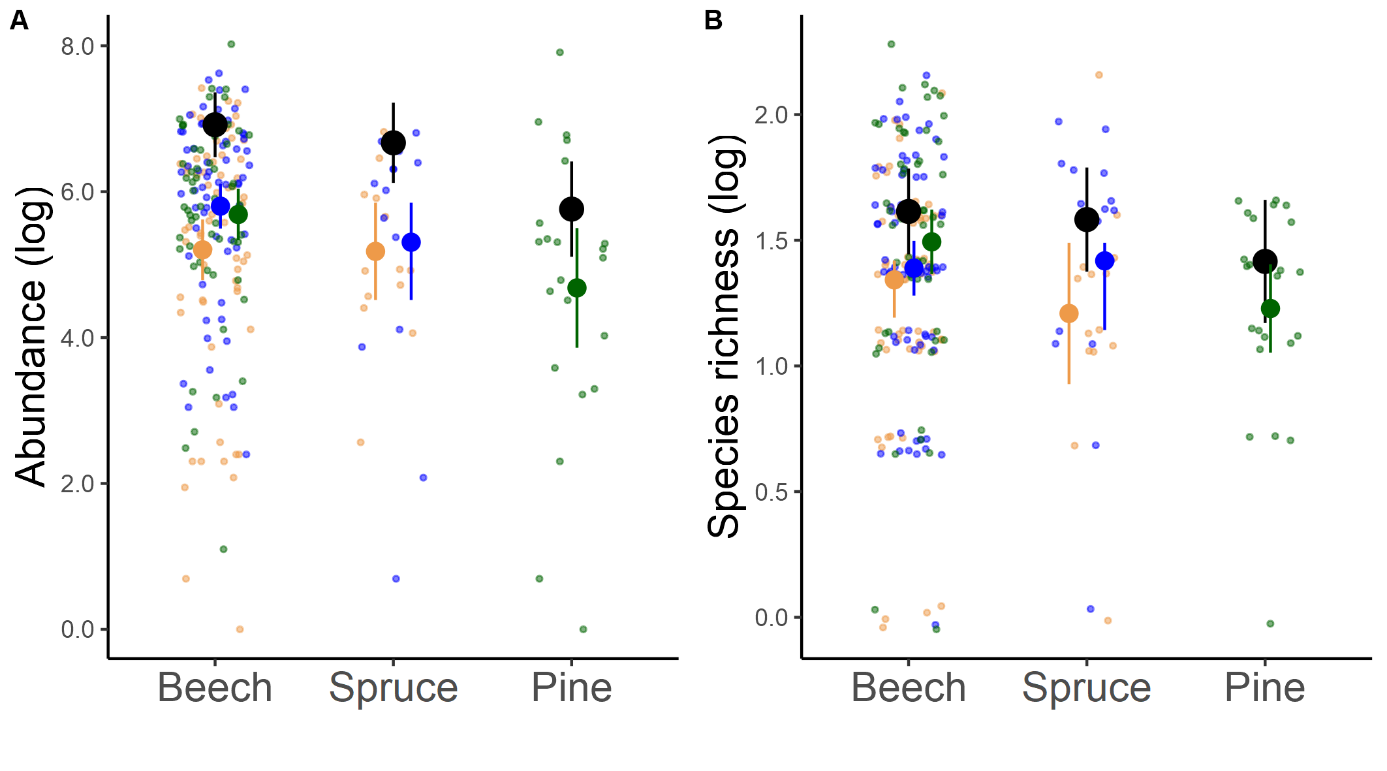


Supporting information Fig. 4. 3D NMDS plot of artificial tree holes based on Bray-Curtis dissimilarity showing the differences in community composition between the plots dominated by beech, spruce and pine trees.


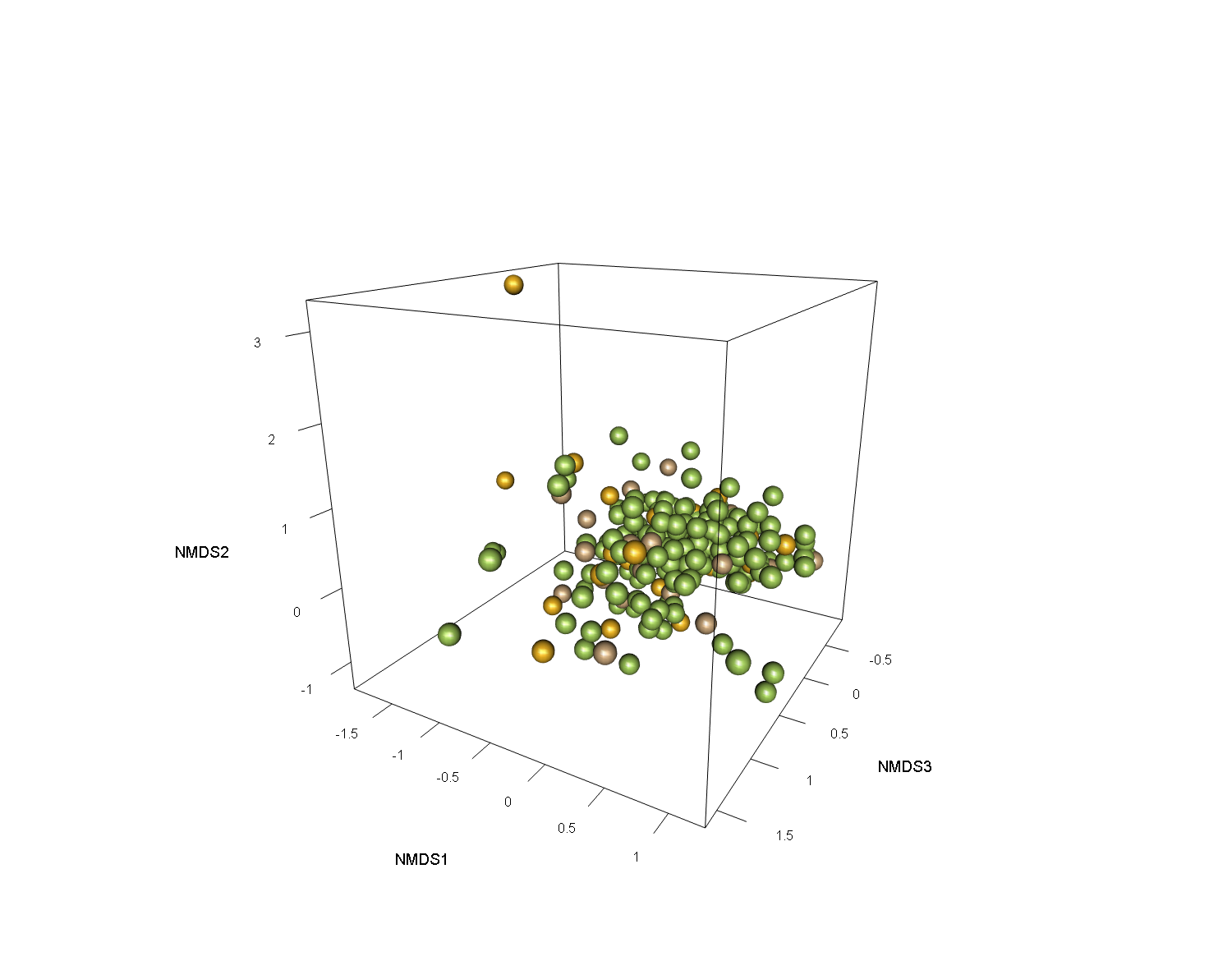


Supporting information Fig. 5. Scanning electron micrographs of larvae of the most abundant and most frequent species *Metriocnemus cavicola* (a), *Dasyhelea* sp. 1(b), and *Myathropa florea* (c). Bars = 0.5 mm.


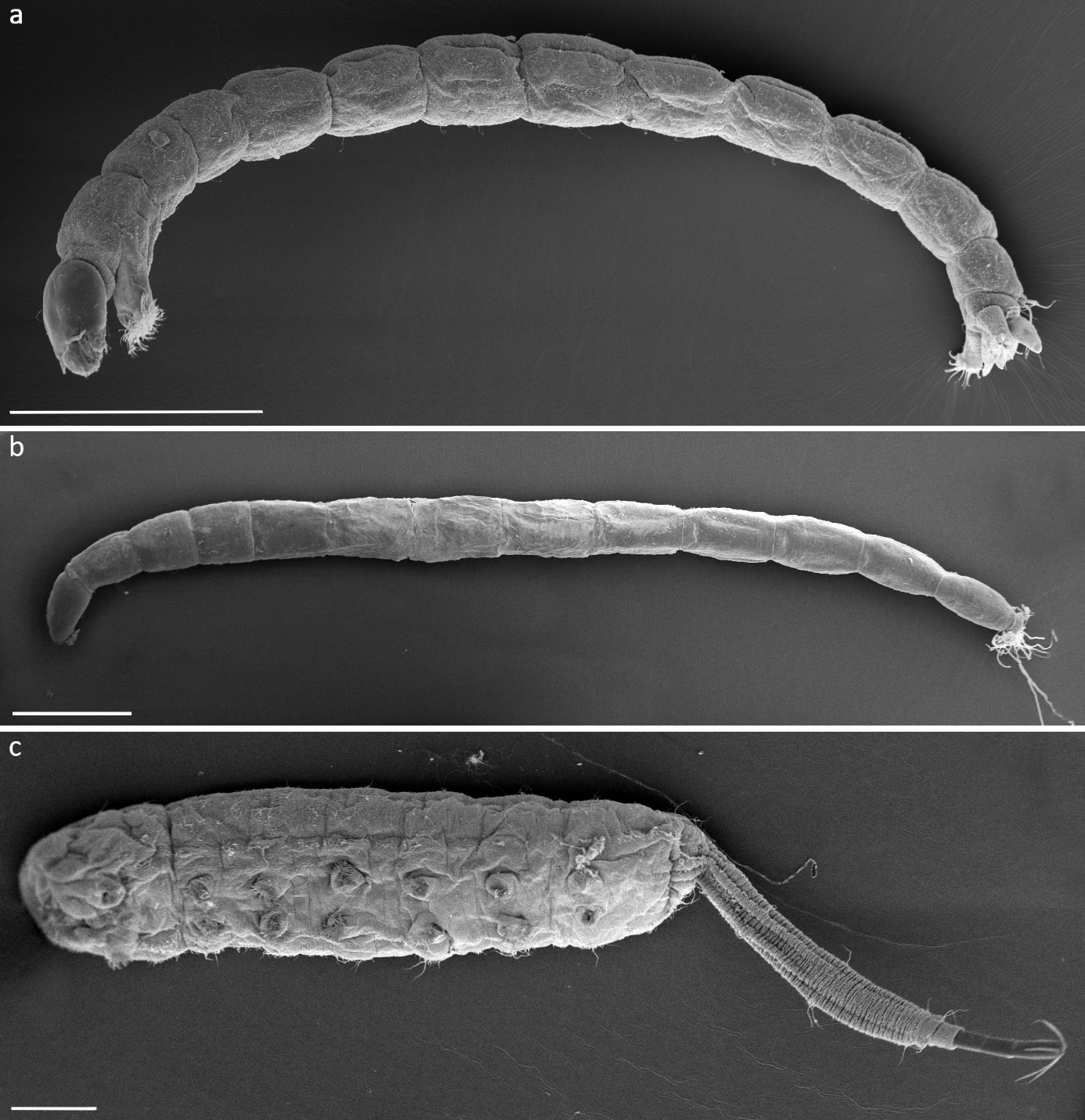


Supporting information Fig. 6. Mean abundance per artificial tree hole of species and morphospecies of the regions Alb (orange), Hainich (blue), and Schorfheide (green).

*
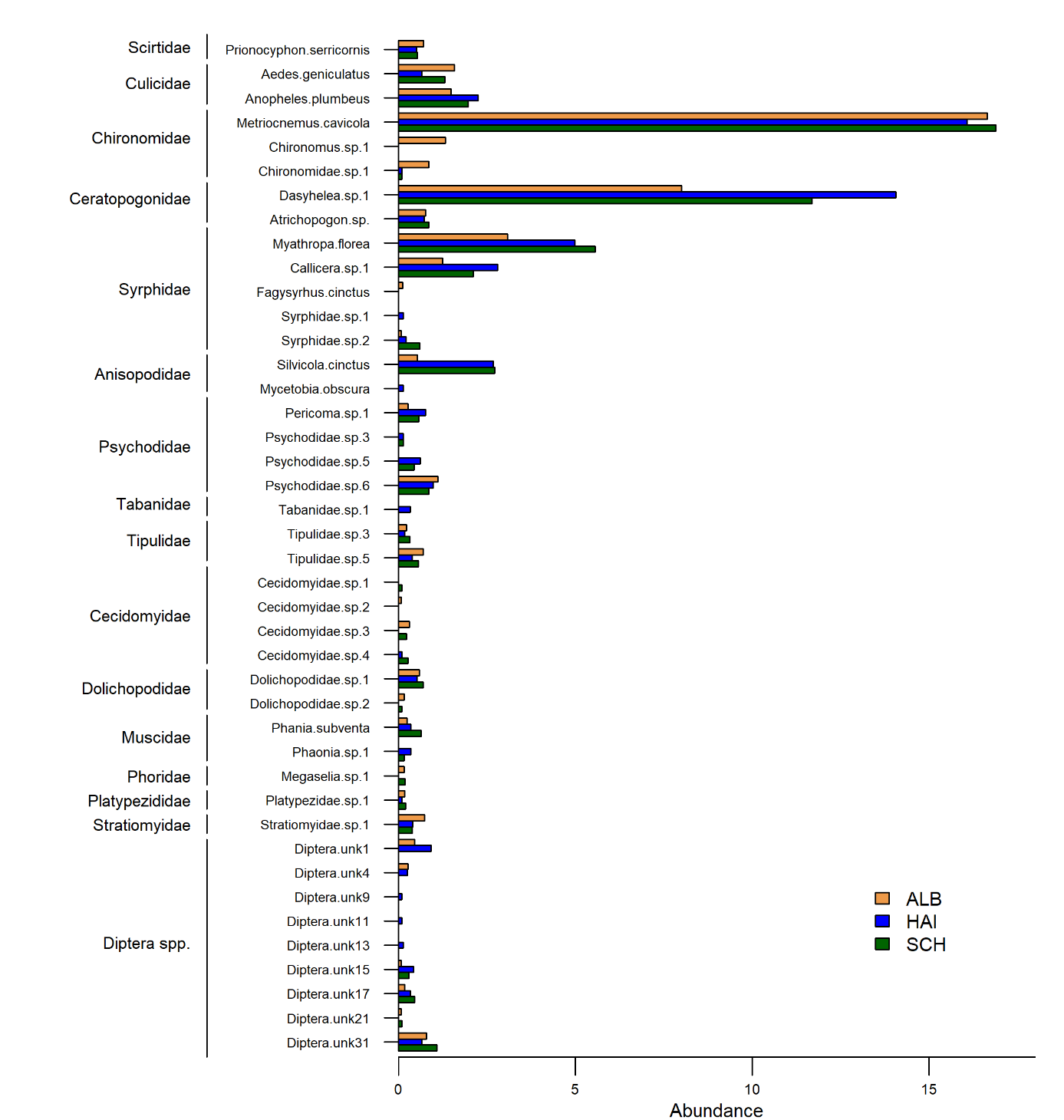
*

Supporting information Fig. 7. Abundance of the three most abundant and most frequent species *Metriocnemus cavicola*, *Dasyhelea* sp. 1 and *Myathropa florea* in the regions Alb, Hainich and Schorfheide. The symbols give the results of the linear mixed models with region as fixed effect and number of tree nested within plot as random factor. Lines show 95% confidence interval. Raw data are plotted as dots for the regions Alb (orange), Hainich (blue), and Schorfheide (green). Abundance data were log-transformed for the analysis, so log-transformed values are also shown here.

*
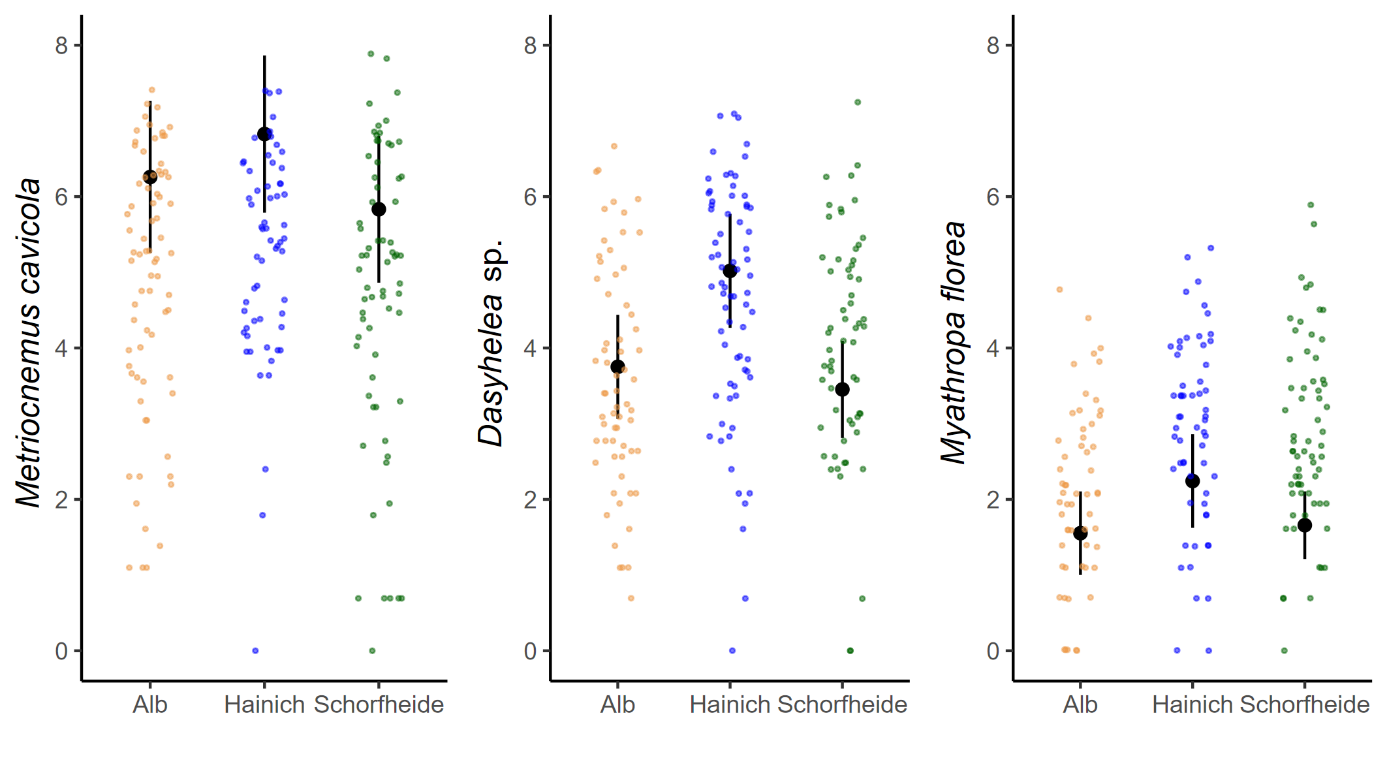
*
